# Editorial: Mind-Brain Plasticity and Rehabilitation of Cognitive Functions: What Techniques Have Been Proven Effective?

**DOI:** 10.3389/fnbeh.2016.00232

**Published:** 2016-12-20

**Authors:** Katiuscia Sacco, Benedetto Sacchetti

**Affiliations:** ^1^Imaging and Plasticity Research Group, Department of Psychology, University of TurinTurin, Italy; ^2^Neuroscience Institute of TurinTurin, Italy; ^3^Department of Neuroscience, University of TurinTurin, Italy

**Keywords:** rehabilitation, plasticity, brain stimulation, training-induced changes, cognitive functions

Contributions to this special issue represent an effective effort toward the understanding of how and to what extent rehabilitation drives neuronal changes, promoting recovery. Table 1 summarizes all these studies.

**Table 1:** **Assessment techniques and types of treatments**.

**Assessment technique**	**Behavioral**	**Behavioral and brain activity**
**TYPE OF TRAINING**		
Behavioral	^*^ Bolognini et al. (spatial and body representations, sensory and motor impairments) ^*^ Pedroli et al. (spatial, virtual reality, neglect) ^◦^ Ciaramelli et al. (memory, PFC lesions) ^◦^ Lévy-Bencheton et al. (vision, HVFD) ^◦^ Livelli et al. (multi-domains, HIV/AIDS NCD) ^◦^ Mattioli et al. (central executive functions, MS) ^◦◦^ Panerai et al. (multi-domains, NCD)	^*^ Sale and Berardi (vision, rodents) ^*^ Dundon et al. (vision, HVFD) ^*^ Mateo et al. (motor, SCI) # Costa et al. (BDNF, executive domains, Parkinson + MCI) # Kamran et al. (fNIRS) ^◦◦^ Sacco et al. (communication, TBI) ^◦^ Tsai and Wang (executive functions, elderly)
Nervous System Stimulation	^◦^ Bisio et al. (motor, PNS, healthy) ^◦^ de Aguiar et al. (language, tDCS, aphasia)	^*^ Pazzaglia and Galli (visuo-motor, movement disorders) ^◦^ Chaieb et al. (motor learning, tNIRS, healthy) ^◦^ D'Agata et al. (motor, rTMS, tDCS, stroke) ^◦^ Sacco et al. (attention, tDCS, TBI)

*BDNF, brain-derived neurotrophic factor; fNIRS, functional near-infrared spectroscopy; HVFD, Homonymous visual field defects; MCI, mild cognitive impairment; MS, multiple sclerosis; NCD, neurocognitive disorder; PFC, prefrontal cortex; SCI, spinal cord injury; TBI, traumatic brain injury; tDCS, transcranial direct current stimulation; tNIRS, transcranial near-infrared stimulation*.

◦ *Individual sessions*.

◦◦ *Group sessions*.

# *Biomarker research; Model development*.

* *Review/Perspective study*.

A series of reviews have dealt with the efficacy of trainings aimed at the treatment of motor (Mateo et al.; Pazzaglia and Galli), visual (Sale and Berardi; Dundon et al.), sensory and (Bolognini et al.) and spatial disorders (Pedroli et al.).

Mateo et al. made an extensive review on motor imagery effectiveness during reach-to-grasp rehabilitation in spinal cord injury patients with tetraplegia. They found that motor imagery of possible non-paralyzed movements improved reach-to-grasp performance by increasing tenodesis grasp capabilities and muscle strength, decreasing movement time and trajectory variability, and reducing the abnormally increased brain activity. Moreover, motor imagery can be used to control brain-computer interfaces that successfully restore grasp capabilities.

Pazzaglia and Galli wrote a perspective study with the aim of translating novel findings in the perceptual and motor domains into the rehabilitation of movement disorders. Visual-motor approaches seem to maximize neural plasticity and lead to greater effect than visual inputs alone: according to the authors, this is because the first involve multiple sensory channels and thus enable individuals to better predict and optimize motor behavior.

Sale and Berardi reviewed interesting findings in adult rodents concerning the possibility of treating amblyopia, a severe visual function impairment which, until recently, has been effectively treated only in children, as there was no known way to foster adult visual cortex plasticity. Among the new proposed intervention strategies, non-invasive procedures based on environmental enrichment, physical exercise or visual perceptual learning appear particularly promising in terms of future applicability in the clinical setting.

Dundon et al. reviewed neuropsychological training methods of visual rehabilitation of homonymous visual field defects. They include “compensation” paradigms, which compensate vision loss by training eye scanning movements, and “restorations” paradigms, which activate residual visual functions by training light detection and discrimination of visual stimuli. The authors propose that both plasticity within peri-lesional spared tissue and changes between networks (i.e., recruiting alternative visual pathways) contribute to recovery.

Bolognini et al. produced a mini-review to show how crossmodal illusions—products of multisensory integration—can be used in rehabilitation settings to restore disarranged spatial and body representations related to pain, sensory, and motor impairments, as well as their use for improving neuroprosthetics.

Pedroli et al. carried out a systematic review of the most recent virtual reality applications for the assessment and rehabilitation of unilateral spatial neglect, providing crucial indications for neurorehabilitation interventions and clinical practice.

Two papers presented biomarker research and model development.

Costa et al. showed a significant positive correlation between brain-derived neurotrophic factor (BDNF) serum levels and cognitive functioning in attention and executive domains in 13 Parkinson's disease patients with mild cognitive impairment. Given the role of BDNF in regulating synaptic plasticity, this trophic factor may be a potential biomarker for evaluating cognitive changes in neurological syndromes associated with cognitive decline.

Kamran et al. working with functional near-infrared spectroscopy (fNIRS), developed an hemodynamic response model able to estimate inter-subject variations in HRF and physiological noises for better cortical functional maps.

A series of research papers looked at behavioral changes as a consequence of behavioral treatments.

Lévy-Bencheton et al. studied 14 left- or right homonymous visual field defect patients with a training based on a single 15 min voluntary anti-saccades task toward the blind hemifield. When combined with an adaptation paradigm, letting automatic sensorimotor adaptation to increase AS amplitude, it improved visual quality of life while exploring visual scenes or reading a text.

Livelli et al. tested a 4 month cognitive rehabilitation protocol on 16 HIV/AIDS patients with HIV-associated Neurocognitive Disorder (HAND), compared to 16 HIV/AIDS without HAND. The experimental group showed cognitive improvements in various domains: those in Abstraction/executive functioning and in Attention/working memory were still present at the 6 month follow up. On the contrary, the control group significantly worsened in the same domains.

Mattioli et al. evaluated the efficacy of a 15 week domain specific cognitive training, compared to a specific psychological intervention, in 41 patients with multiple sclerosis, showing that, at 2 years follow up, patients' submitted to the specific training improved in the majority of the cognitive tests and ameliorated their perceived cognitive performance.

Ciaramelli et al. tested the Preview-Question-Read-State-Test method, a technique used to enhance long-term memory when reading a text, in 7 patients with mild memory problems due to prefrontal cortex lesions, showing that it improves immediate and delayed recall, as well as the ability to answer questions of comprehension. The same improvement is present both when the experimenter formulated the questions about the text, and when the patients did it on their own.

Panerai et al. carried out a daily group Intensive Cognitive Activation protocol, over a period of 2 months, in 16 patients with major neurocognitive disorder (NCD) and 15 with mild NCD; a control group of 11 patients with major NCD was used as a control group. General cognitive functioning and other specific functions, including attention, ideomotor praxis and visual memory, improved in all patients. Besides, while long- and short-term verbal memory worsened in controls, they did not in the experimental groups.

Two research papers looked at behavioral changes as a consequence of nervous system stimulation.

Bisio et al. showed, on 48 healthy participants, that spontaneous movement tempo—the movement freely produced by subjects tapping out a rhythm with their fingers—can be modified by action observation (AO) combined with peripheral nerve stimulation (PNS), even in absence of immediate movement execution. The induced changes in spontaneous movement tempo, attributable to neuroplasticity mechanisms, indicate possible application of AO-PNS in rehabilitative treatments.

de Aguiar et al. showed that a linguistically-motivated language therapy focusing on verb inflection and sentence construction, combined with transcranial Direct Current Stimulation (tDCS), is effective in producing both item-specific and generalized improvement in 9 individuals with post-stroke aphasia.

Two research papers looked at behavioral and brain activity changes as a consequence of behavioral treatments.

Sacco et al. proposed a group training program for the rehabilitation of communicative abilities and tested it on 8 traumatic brain injury patients, showing an improvement in overall communicative performance which was still present 3 months later. Besides, they found increased amplitude of low frequency fluctuation, measured through resting state functional magnetic resonance imaging (fMRI), in brain regions involved in communication.

Tsai and Wang studied 64 elderly individuals, divided in three groups, showing that physical exercise reduces reaction times during a task-switching paradigm with unpredictable and infrequent switches, and it enhances electrophysiological response related to executive functioning (P2 and P3 amplitudes). It seems that open-skill exercise has a small advantage on executive control with respect to closed-skills.

Finally, three papers dealt with behavioral and brain activity changes as a consequence of brain stimulation treatments.

Chaieb et al. studied the neuroplastic effects of transcranial near-infrared stimulation (tNIRS) on the motor cortex in healthy participants. The serial reaction time task was used to investigate the possible effect of tNIRS on implicit learning. A significant decrease in the amplitude of motor-evoked-potentials (MEPs) was observed up to 30 min post-stimulation. Furthermore, the short interval cortical inhibition was increased and facilitation decreased significantly after tNIRS. Such results have to be taken into account when using tNIRS to elicit plastic changes in TBI or stroke patients.

D'Agata et al. used Transcranial Magnetic Stimulation (rTMS) and transcranial Direct Current Stimulation (tDCS), for upper limb rehabilitation of 34 patients with stroke, showing that the effects of the two techniques are comparable, with some advantages using tDCS versus rTMS. They also found that more than one cycle (2–4 weeks), spaced out by washout periods, should be used, only in responder patients, to obtain clinical relevant results.

Sacco et al. showed that 10 sessions of transcranial direct current stimulation (tDCS), each followed by computer-assisted training, improved divided attention in 16 traumatic brain injured (TBI) patients, compared to 16 TBI for whom the training was preceded by sham stimulation.

Functional magnetic resonance imaging (fMRI) data showed neural changes, interpreted as normalization of previously abnormal hyperactivations.

## Author contributions

KS drafted the Editorial and BS approved it for publication.

### Conflict of interest statement

The authors declare that the research was conducted in the absence of any commercial or financial relationships that could be construed as a potential conflict of interest.

